# Utilizing the Fujimori Gate Flap for the Reconstruction of Squamous Cell Carcinoma: A Case Report

**DOI:** 10.7759/cureus.65348

**Published:** 2024-07-25

**Authors:** S. Vandana Ajay, Sanjana N Wadewale, Chetan Gupta, Shreya S Pawar, Nitin Bhola, Vaibhav Pipare, Tikeshwari Gurav

**Affiliations:** 1 Department of Oral and Maxillofacial Surgery, Sharad Pawar Dental College and Hospital, Datta Meghe Institute of Higher Education and Research, Wardha, IND; 2 Department of Prosthodontics, Crown and Bridge, Sharad Pawar Dental College and Hospital, Datta Meghe Institute of Higher Education and Research, Wardha, IND; 3 Department of Endodontics, Sharad Pawar Dental College and Hospital, Datta Meghe Institute of Higher Education and Research, Wardha, IND

**Keywords:** fujimori gate flap, lip reconstruction, excision, surgical management, squamous cell carcinoma

## Abstract

The surgical treatment of oral squamous cell carcinoma (SCC) results in tissue defects caused by the removal of the cancerous tissue. There are various reconstruction options available for lip construction. Harvesting the flap to reconstruct these defects undoubtedly results in substantial morbidity. Lip reconstruction can be performed more efficiently and with reduced side effects by utilizing flaps, which can minimize donor site morbidity and shorten surgical harvesting time. We are reporting a case involving a 52-year-old male with SCC of the lip who presented without any comorbidity. This case report describes the careful lip reconstruction using the Fujimori gate flap technique following complete surgical excision of the lesion.

## Introduction

Two relatively prevalent cancers that afflict areas exposed to the sun are squamous cell carcinoma (SCC) and basal cell carcinoma. While basal cells are often found in the upper lip and SCCs in the lower lip, lip carcinomas frequently exhibit a classic, distinct distribution pattern.

As one ages, the incidence rises, peaking in the seventh and eighth decades of life. Compared to the upper lip (1%-15%), the lower lip (80%-95%) is more often affected [1]. The proportion of males to females affected by lip cancer is around 15:1. Individuals with fair skin tones and those with prolonged sun exposure are the most prevalent causes of lip cancer. In 2020, there were 377,713 reported oral oral SCC (OSCC) cases worldwide. The Global Cancer Observatory predicts that, by 2040, the incidence of OSCC will increase by around 40%, leading to a corresponding rise in mortality [2].

The causes of lip cancer include a variety of multifactorial factors, including work-related exposure, smoking, UV radiation, trauma, illnesses, and some endogenous factors [2]. While the clinical manifestations of SCC, such as erythematous patches and nodules, are diverse, the typical presentation is as indurated, well-demarcated ulcerative lesions. Although SCC can develop in normal skin, it most commonly arises from preexisting cutaneous lesions like solar keratosis, leukoplakia, and radiodermatitis [3].

With early diagnosis and appropriate treatment planning, prognosis and cure rates have steadily risen. Surgery is a problem when reconstructing, particularly for advanced and extensive lesions [2]. Maximal oral aperture, oral competence, and cosmesis preservation are the aims of lip reconstruction. We present a case where we used the Fujimori gate flap to reconstruct a lower lip defect in a case of well-differentiated SCC.

## Case presentation

**Figure 1:**
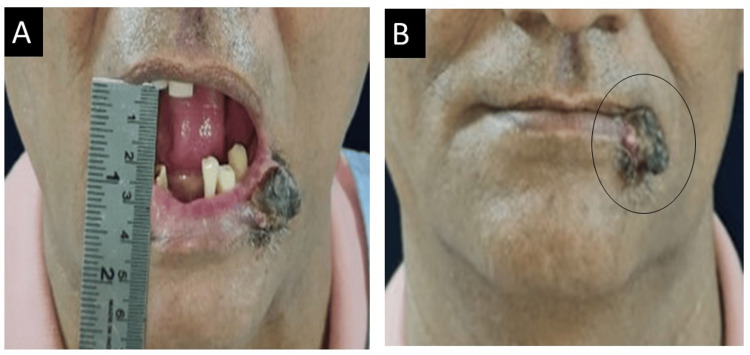
Extent of ulceroproliferative growth over the lower lip. (A) Mouth opening. (B) Front view

A 49-year-old male patient reported to the Department of Oral and Maxillofacial Surgery, Sharad Pawar Dental College and Hospital, Sawangi, Wardha, with the chief complaint of a nonhealing ulcer over the lower lip for six months. Initially, he started noticing a small pea-sized, nonhealing ulcer on his lower lip, which gradually increased to its current size of 2.5 cm × 2 cm approximately, as shown in Figure 1.

Upon assessment, the patient presented with sudden onset, localized pain exacerbated by the consumption of hot and spicy foods and mastication, and subsequently relieved spontaneously. The patient reported a history of kharra and tobacco chewing in the last 15-20 years, 8-10 times a day approximately. Six months ago, the patient sought care at a private hospital in Paratwada for the same chief complaint, where an incisional biopsy revealed a diagnosis of well-differentiated squamous cell carcinoma. The patient did not seek treatment and reported to the outpatient department in Sawangi. On clinical examination, the ulcer was roughly oval and ill-defined, with diffuse borders, everted margins, and an irregular surface. On palpation, tenderness and induration were present, and consistency was firm. A single palpable lymph node in the right submandibular region, measuring 1 cm × 0.5 cm, was roughly oval, mobile, nontender, and soft in consistency. Along with the history of pain, bleeding was also present on touch. There was no significant medical or dental history.

Looking into the clinical scenario, the provisional diagnosis was carcinoma of the lower lip. Confirmation by incisional biopsy revealed a report that suggested well-differentiated lower lip squamous cell carcinoma. The patient received advice for blood investigations, revealing that their complete blood count, hepatitis B surface antigen, and hemoglobin A1c levels fell within normal ranges, and the HIV test yielded a nonreactive result.

**Figure 2:**
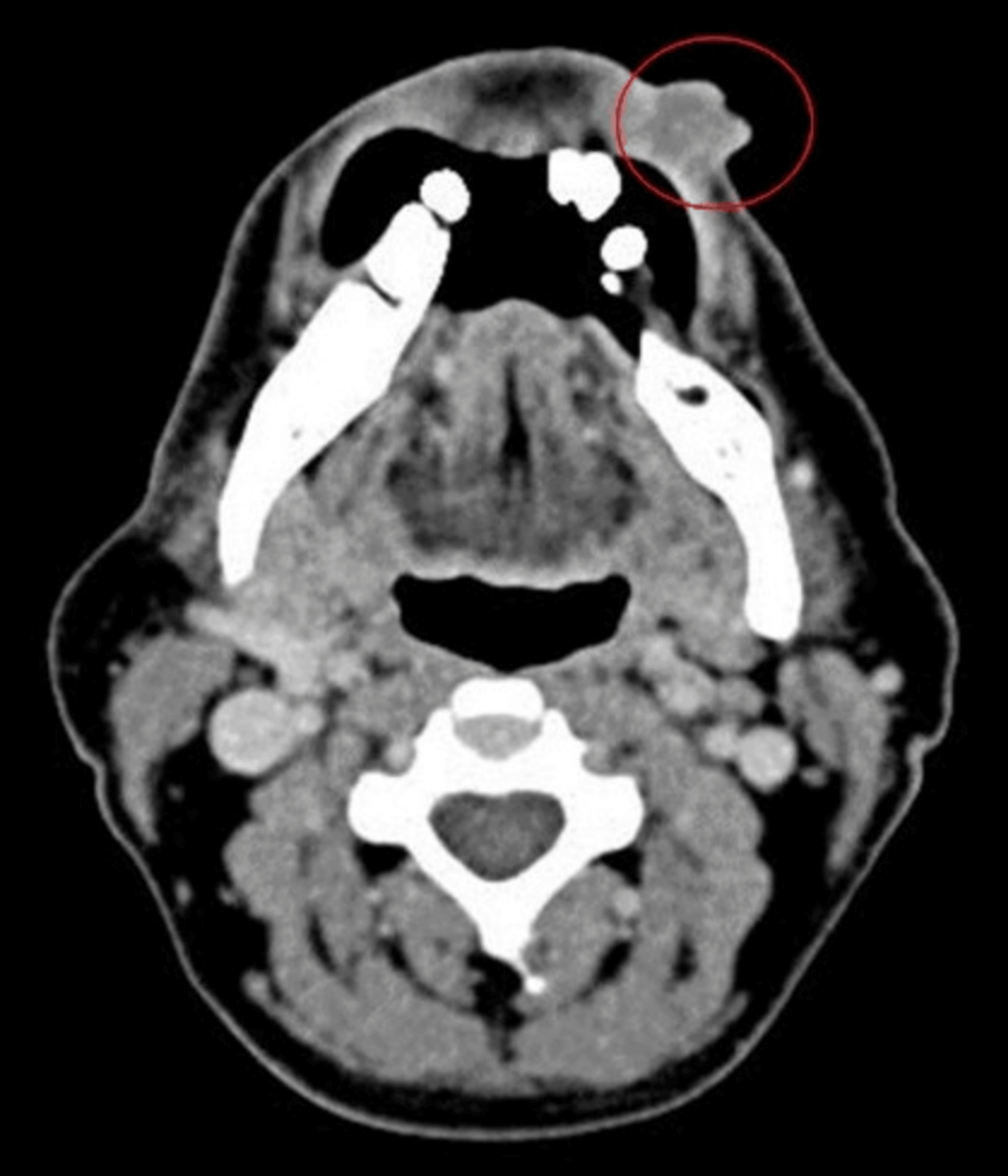
Cross-sectional CECT image CECT: contrast-enhanced computed tomography

A radiological modality, i.e., a contrast-enhanced computed tomography (CECT) scan of the head and neck, was used to see the extension of the lesionsignificant medical or dental . The axial T1-weighted CECT image reveals a prominently hyperdense mass distinctly involving the lower lip, as shown in Figure 2.

**Figure 3:**
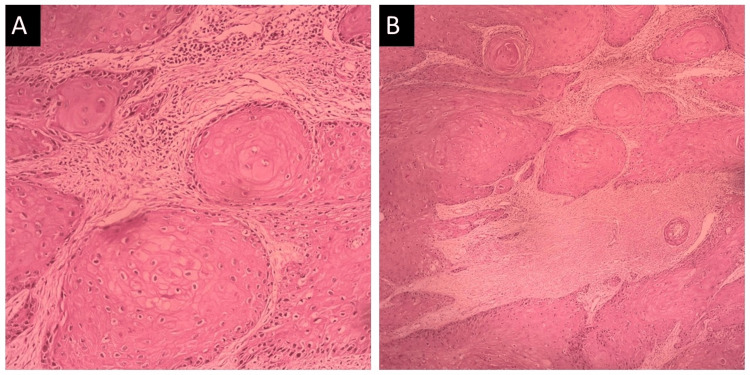
Histopathological examination under (A) low-power microscope (4×) and (B) high-power microscope (10×)

Under local anesthesia, an incisional biopsy was performed, and a histopathological test was conducted, where the specimen was stained with hematoxylin and eosin. Upon thorough histopathological analysis of the specimen, under the magnification of 4× and 10×, the examination revealed the presence of keratin pearls, which are concentrically arranged aggregations of keratinized cells alongside islands of epithelial cells dispersed throughout the fibrous connective tissue stroma. Furthermore, notable observations included variations in the cytoplasmic architecture, characterized by changes in granularity and vacuolation, as well as nuclei exhibiting prominent nucleoli and irregular chromatin patterns, as shown in Figure 3. Based on all investigations and history given by the patient, a final diagnosis of well-differentiated squamous cell carcinoma of the lower lip was made.

**Figure 4:**
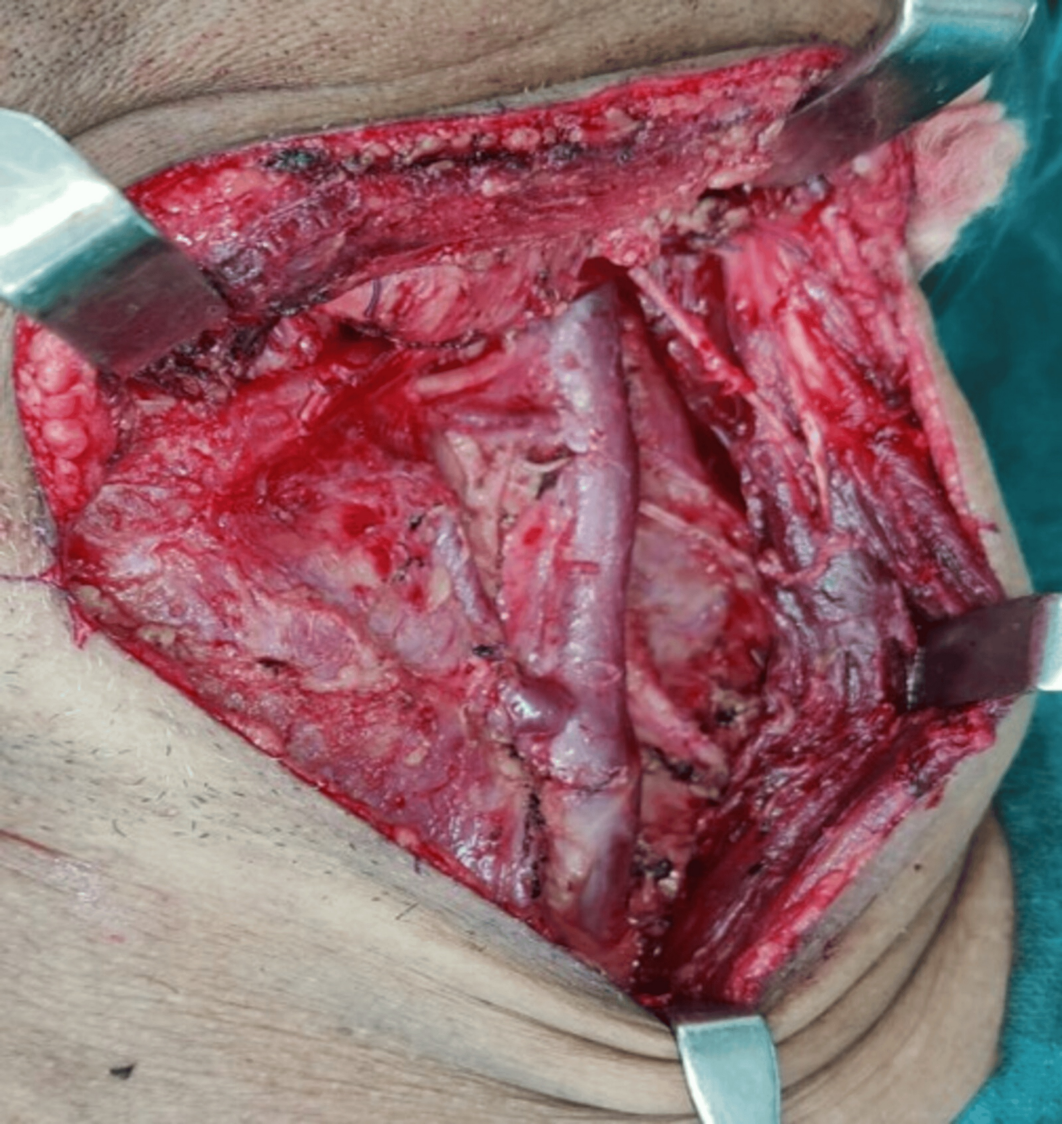
Presentation of the neck dissection procedure

After thoroughly evaluating the extent of the disease, we have meticulously created a thorough surgical strategy. This comprehensive plan encompasses performing a neck dissection, as shown in Figure 4.

**Figure 5:**
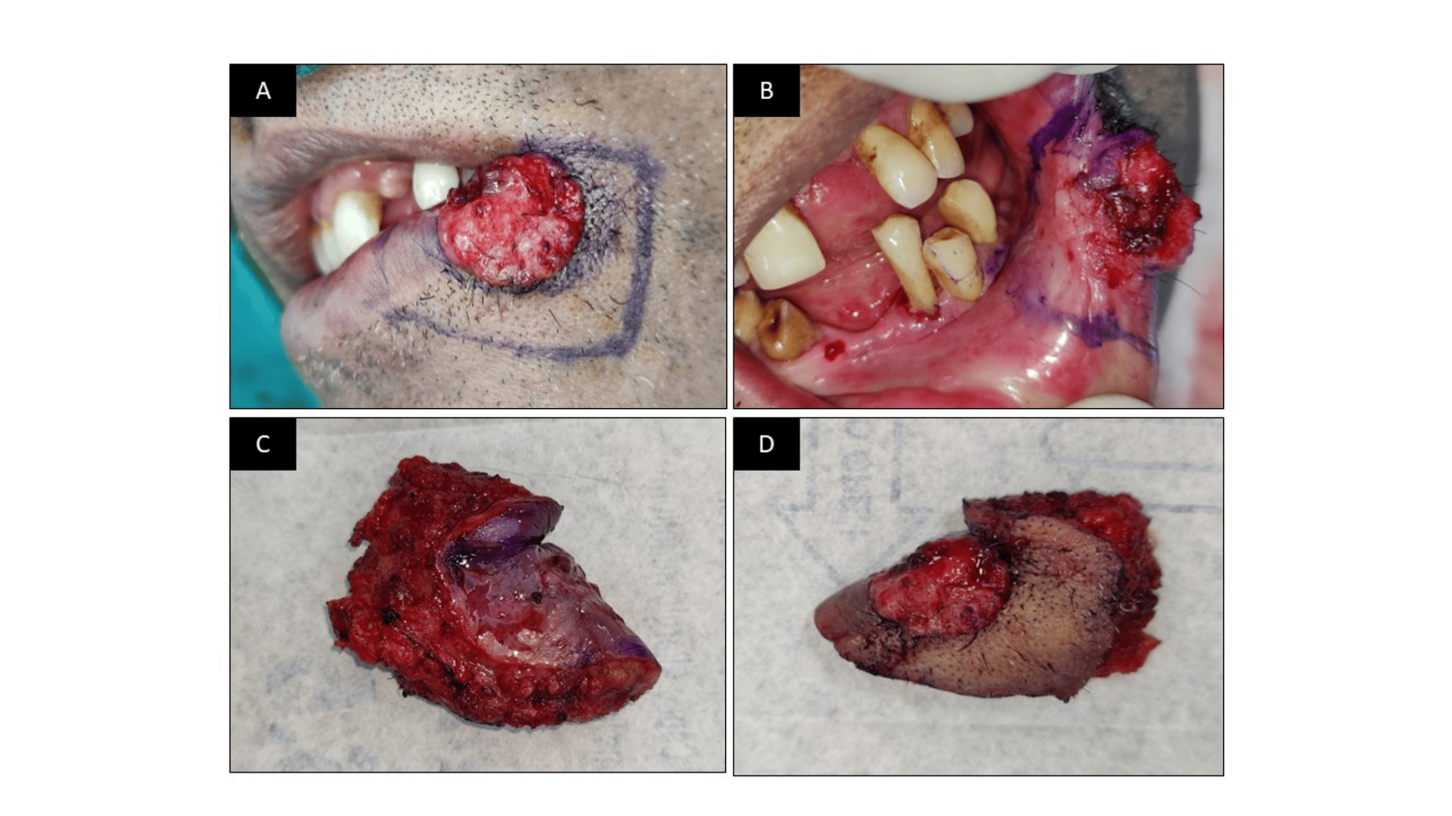
Excision of the ulcer. (A) Extraoral marking. (B) Intraoral marking. (C,D) Excised tissue

Following neck dissection, the area to be excised was marked surrounding the lesion, and a wide local excision was performed to remove the lesion. After the excision of the lesion, the surrounding tissue was excised from the corner of the lip to one-third of the lower lip, as shown in Figure 5.

**Figure 6:**
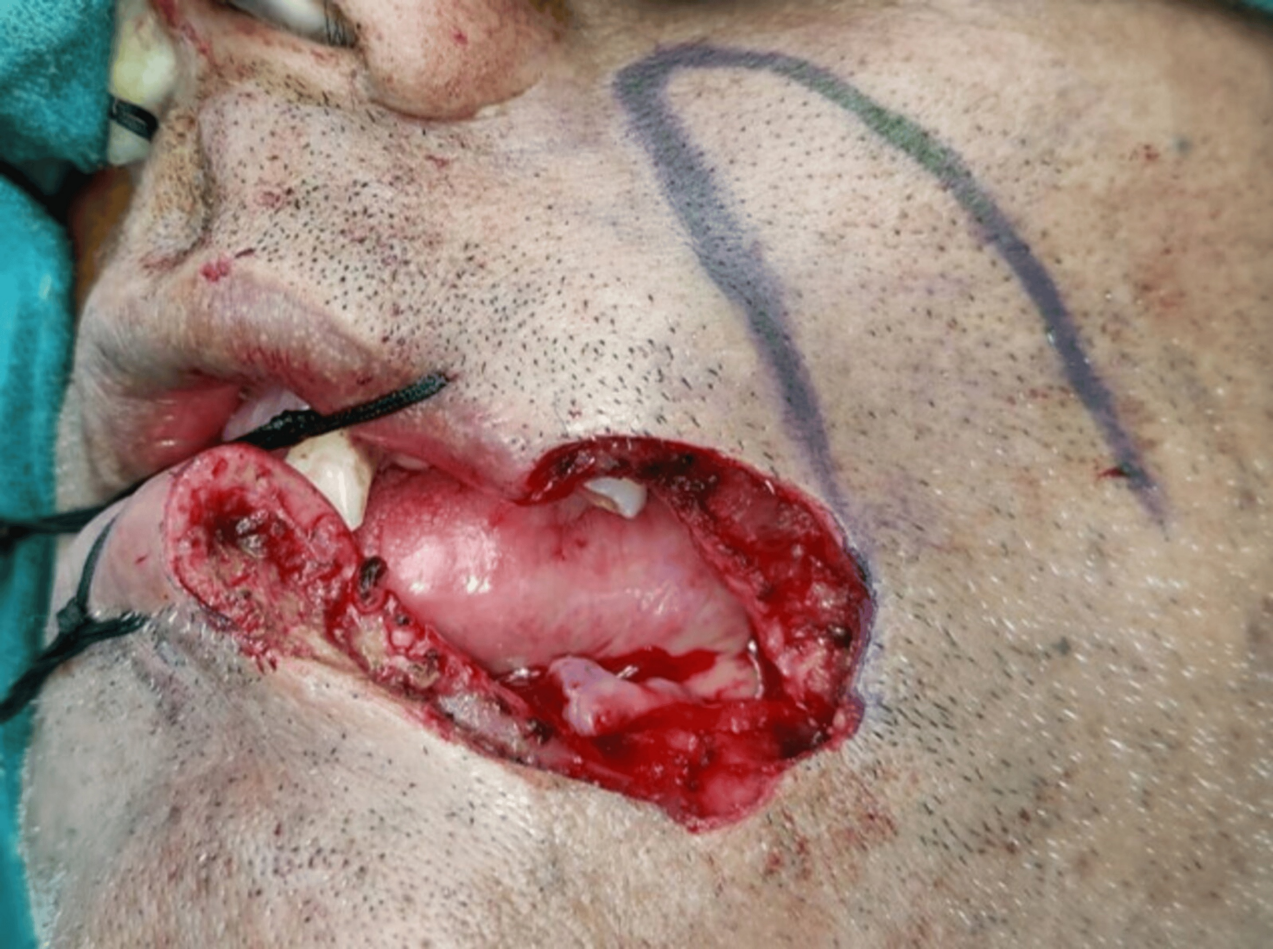
Fujimori gate flap marking

The surgeon meticulously marked the precise area for the Fujimori gate flap and then made a careful incision along the marked lines. After gently lifting the flap, the lower lip was reconstructed using the Fujimori gate flap under general anesthesia, as shown in Figure 6.

**Figure 7:**
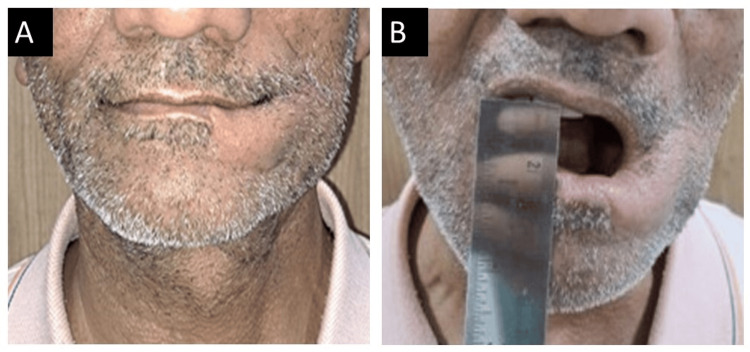
(A,B) Postoperative aspect of the patient after six months of follow-up

After six months, the patient's subsequent appointment was scheduled. The patient showed no evidence of disease, as shown in Figure 7.

## Discussion

The main therapeutic approaches that strive to achieve both functional and esthetic results are tumor excision, neck dissection, and reconstruction. Surgery can be used to treat lower lip cancer by removing primary lesions with a tumor-free margin. The wider extent of the lesion of lower lip length may make it challenging for the surgeon to reconstruct. The surgeon is guided by several considerations while deciding on the best reconstructive method [2].

When a defect is restricted to the lip vermillion, a mucosal flap from the left lip covers the underlying muscle to prevent scarring. Other options include using tissue flaps from the underside of the tongue or the opposite lip or using V-Y advancement if the gap is too big to be closed with a simple approach [1].

Primary closure is an option for abnormalities affecting less than one-third of the lip's breadth in full thickness. A lip defect can be primarily closed using a variety of excision techniques.

The Abbe flap and the Abbe-Estlander flap are utilized for more significant flaws [2]. The recommended approach is to repair full-thickness abnormalities involving one to two-thirds of the lip, as long as the oral commissure remains intact. This type of repair can be achieved by creating a full-thickness flap using either the upper or lower lip. The Abbe flap offers an effective solution for adjusting the vertical height of the defect, and vermillion tissue can be imported [3,4]. It is mostly advised for correcting anomalies involving the lower lip, such as the commissure. Typically, this course of therapy narrows the commissure, requiring a second-stage commissuroplasty. This treatment involves a lateral triangle incision at the modiolus and is often performed 12 weeks later [5,6].

Another excellent flap option for reconstructing the lower lip deformity is the karapandzic flap. The potential to undertake a single-stage reconstruction of massive full-thickness lower lip abnormalities and the great cosmetic result of the incisions being made in the nasolabial and mentolabial skin creases are the main benefits of the karapandzic flap. It is perfect for lateral anomalies affecting the commissure and for rectangular lesions of the middle lower lip. Moreover, it could be helpful for those who have had radiation therapy in the past or for those who have a weakened blood supply [7].

The Gillies fan flap is a highly effective single-stage technique for reconstructing substantial defects [8,9]. When carried out bilaterally, this technique can be utilized to rebuild full-thickness central lip deformities and lateral whole or nearly total lip abnormalities. Even though flap advancement may cause the commissure to disappear and the lip to shorten (microstomia), both have a negative esthetic impact.

In 2003, Aytekin et al. employed the Fujimori gate flap technique for upper lip defect reconstruction in a patient diagnosed with upper lip carcinoma [10]. We employed a Fujimori gate flap for lower lip restoration, considering the patient's health, specific needs, local tissue characteristics, and the size and location of the defect. Fujimori gate flap has various pros, such as the mucosal flaps providing good vermillion coverage and keeping the facial vessels intact. However, this flap may often require revisional surgeries, and there is often the chance of upper lip denervation. The Fujimori procedure involves making two triangular, approximately 3-cm-wide nasolabial compound island flaps on the cutaneous side [11,12].

The interior of a flap created by mucosal incisions will be wider by approximately 4 cm; this mucosal excess is required for vermilion restoration. The muscle and mucosa remain intact because the incisions are restricted to the skin and subcutaneous tissue [13]. Subsequently, the flaps turned into the defect area and were activated. The suture lines are positioned intraorally along the vestibular sulcus, at the vermilion edge, the labiomentonier groove, and the nasolabial fold, in that order. Partially restoring sensitivity and motor function involves activating the innervated cheek muscles and the mucosal lining over the lower lip. Nearly comparable in color and texture to the native lower lip is the nasolabial tissue employed in this reconstructive technique [14].

## Conclusions

We observed a 49-year-old male patient with a major risk factor of kharra and tobacco chewing. The lower lip is the primary site for cancer of the lip, which affects men mostly and has an around 90% overall survival rate. It was discovered that the initial tumor's size, histologic grade, and involvement subsite all had an impact on the very rare occurrence of cervical lymph node metastasis. Surgery, radiation therapy, chemotherapy, and their combinations are among the possible treatments for lip SCC. For the majority of patients with squamous cell carcinoma of the lip, we recommended surgical tumor removal followed by repair of the lip deformities. Every patient in this series received the surgical procedure. The primary objective of the procedure is to remove the tumor entirely and to provide a safe surgical margin. The skin, muscles, and underlying mucosa must all be fully thickened. Prompt neck treatment and thorough follow-up are still crucial measures. SCC of the lip can only be successfully treated with early identification.
